# A laboratory study of hydraulic fracturing at the brittle-ductile transition

**DOI:** 10.1038/s41598-021-01388-y

**Published:** 2021-11-16

**Authors:** Francesco Parisio, Keita Yoshioka, Kiyotoshi Sakaguchi, Ryota Goto, Takahiro Miura, Eko Pramudyo, Takuya Ishibashi, Noriaki Watanabe

**Affiliations:** 1 Chair of Soil Mechanics and Foundation Engineering, Technische Universitaet Bergakademie, Freiberg, Germany; 2 Department of Environmental Informatics, Helmholtz Centre for Environmental Research - UFZ, Leipzig, Germany; 3 Department of Environmental Studies for Advanced Society, Graduate School of Environmental Studies, Tohoku University, Sendai, Japan; 4 Fukushima Renewable Energy Institute, National Institute of Advanced Industrial Science and Technology (AIST), Koriyama, Japan

**Keywords:** Geophysics, Hydrogeology, Volcanology, Mechanical properties, Geothermal energy, Mechanical engineering

## Abstract

Developing high-enthalpy geothermal systems requires a sufficiently permeable formation to extract energy through fluid circulation. Injection experiments above water’s critical point have shown that fluid flow can generate a network of highly conductive tensile cracks. However, what remains unclear is the role played by fluid and solid rheology on the formation of a dense crack network. The decrease of fluid viscosity with temperature and the thermally activated visco-plasticity in rock are expected to change the deformation mechanisms and could prevent the formation of fractures. To isolate the solid rheological effects from the fluid ones and the associated poromechanics, we devise a hydro-fracture experimental program in a non-porous material, polymethyl methacrylate (PMMA). In the brittle regime, we observe rotating cracks and complex fracture patterns if a non-uniform stress distribution is introduced in the samples. We observe an increase of ductility with temperature, hampering the propagation of hydraulic fractures close to the glass transition temperature of PMMA, which acts as a limit for brittle fracture propagation. Above the glass transition temperature, acoustic emission energy drops of several orders of magnitude. Our findings provide a helpful guidance for future studies of hydro-fracturing of supercritical geothermal systems.

## Introduction

The quest to exploit geothermal energy directly from the roots of volcanic systems^1,2^ is raising new issues about the role played by temperature on traditional reservoir engineering processes. A common assumption of an essentially impermeable crust above $400\,^{\circ }$C^3^ has been challenged by laboratory findings^4,5^ and in-situ observations^6,7^. Nonetheless, harvesting energy from so-called supercritical geothermal systems^8^ might require permeability enhancement and it is unclear whether or not the current stimulation techniques are directly applicable in reservoirs where $T\ge 450\,^{\circ }$C. Experimental work on laboratory rock samples has shown that micro-fracturing occurs during injection as fluid percolation reduces the effective stress and creates tensile states in the rock^5,9^. What is at present not yet fully understood, is the role played by the rheology (we intend rheology as the science that studies the deformation of matter) of the rock in high-temperature hydro-fracturing scenarios. Our contribution provides insights on the influence of the deformation mode on the propagation of hydraulic fractures.

Hydro-fracturing and dike propagation laboratory experiments are often performed with the aid of rock or fluid proxies at a lower and more controllable temperature and/or pressure^10^. To this end, transparent manufactured materials such as polymethyl methacrylate (PMMA) or Polyurethane (PU) are a common choice to study hydraulic fracturing^11–14^. The transparency of PMMA and PU facilitates the direct observation of the fracturing behavior and the mechanical properties are well characterized or can even be tailored to specific experimental requirements^15^. For example, solid PMMA has been used as a rock-analogue to experimentally validate the crack tip behaviors predicted by the hydraulic fracturing theory in penny-shape^16^ and PKN geometry^17^, and molten PMMA has been used as a magma-analogue in dike propagation experiments^18^. The transition between brittle and ductile deformation mode in PMMA occurs approximately in the range $80-110\,^{^\circ }$C^19–21^, which provides an essential advantage as a rock-analogue at the the brittle-ductile transition. Although experimental apparatuses can reach a temperature close to the ductile transition of certain rocks^4,22,23^ and have been previously employed to study water-based supercritical hydraulic-fracturing^5^, testing at lower temperature conditions implies that the propagating fluid (water) is still in its liquid state; in combination with the low permeability of PMMA, it allows to separate the effects of pure solid rheology from the ones of low-viscosity fluid percolation.

**Figure 1:**
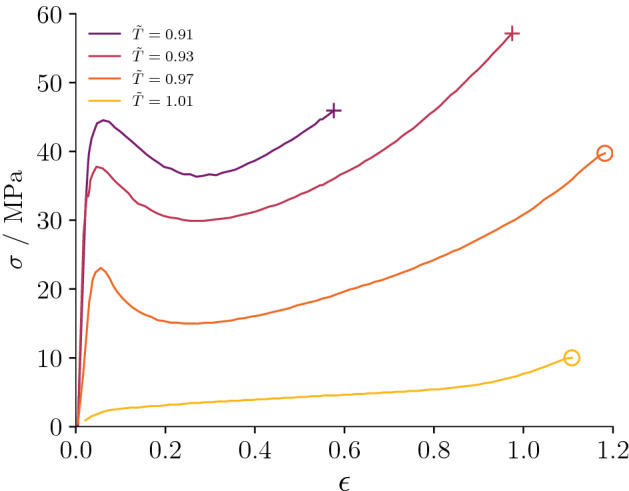
Uniaxial stress $\sigma$ vs strain $\varepsilon$ in PMMA under tension loading (digitized from^21^): ductility increases with temperature while the whole rheology drastically changes across the glass transition regime^21^. The plus symbol at the of the curves represents final fracture propagation, while the open dots implies that no final fracturing occurred.

The rheology of PMMA shifts from brittle toward ductile deformation and visco-plastic flow conditions as the temperature increases and the strain rate decreases^21^. In the cold and brittle regime, approximately at ambient temperature, crazing (i.e., the formation of tensile microvoids^24^) and fracturing are the dominating failure mechanisms, while with increasing temperature, failure occurs through plastic yielding and shear localized deformation^20^. Above the glass transition temperature $T_\text{g}$, the deformation is dominated by visco-plastic ductile flow and a plateau in the rheological properties is observed at the rubbery regime^21^. As temperature increases beyond the rubbery regime, a further collapse of stiffness occurs at the viscous flow regime, where the material behaves as a viscous melt^21^. Figure 1 shows the drastic change in rheology of PMMA across the glass transition temperature. At ${\tilde{T}}=T/T_\text{g}<1$, the uniaxial stress $\sigma$ vs uniaxial strain $\varepsilon$ curves are self-similar. Ductility increases towards ${\tilde{T}}=1$ and at ${\tilde{T}}>1$ softening, peak-stress and stress bearing capacity disappear, marking the strong rheological transition typical of polymers.

In this manuscript, we investigate the hydraulic-fracturing propagation across the brittle-ductile transition of PMMA. Our study assumes PMMA as an analogue of rock in which flow through and equations of state of the propagating fluid play a negligible role, such that the rheological features of the solid can be isolated from the effects occurring in permeable and fractured rocks at the supercrtical temperature of water. The detail of the experimental apparatus and program employed in the study can be found in the “Methods” section. There, we briefly review the constitutive theory of PMMA and introduce a simple plastic-damage model to compute fracture initiation in complex 3D conditions with non-uniform loading and the effects of a finite-length well. In particular, two procedures are devised to highlight the effects of non-uniform conditions: (i) a first procedure called P1, in which injection occurs within a borehole that extends half-way through the samples, a non-symmetric o-ring is placed on the wellbore’s intersection face and the load path is designed to maximize the initial deviatoric stress; (ii) a second procedure called P2, in which injection occurs within a borehole that fully extends through the samples, a symmetric o-ring is placed on the wellbore’s intersection face and the load path is designed to minimize the initial deviatoric stress. Procedure P1 maximizes stress concentrations and promotes non-planar fracture growth, while procedure P2 enforces pure mode-I propagation (see Methods for a detailed description of P1 and P2). The manuscript discusses the results from the experimental program in terms of injected fluid pressure, acoustic emissions (AE), deformation and post-mortem images of the PMMA samples. We interpret the experimental results with the aid of numerical modeling. Below the glass transition temperature, we observed an increase in ductility during hydro-fracturing up to ${\tilde{T}}\le 0.96$, while no hydro-fracture propagation is observed at ${\tilde{T}}\ge 0.99$: this sets the limit of hydro-fracturing practically at the glass transition temperature. In the discussion section, we assess the implications for hydro-fracturing and stimulation in supercritical and other high temperature geothermal reservoirs and the possible directions of future investigations.

## Results

### Stress concentrations effect

**Figure 2:**
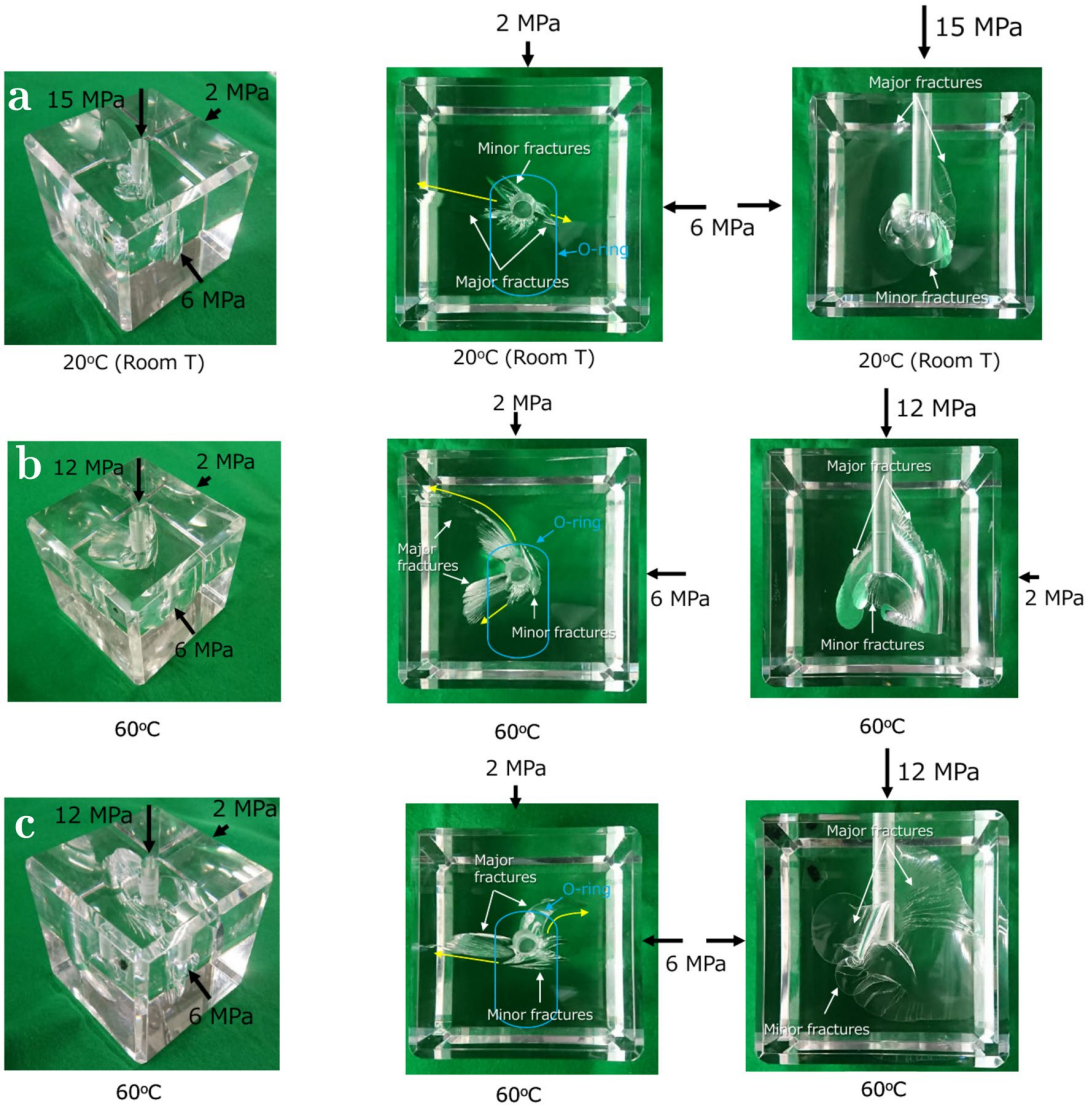
Images of the post-mortem samples tested following procedure P1 (see Methods) at $20\,^{\circ }$C (**a**) and twice at $60\,^{\circ }$C (**b** and **c**) for repeatability. The ensemble of mode-I and mixed-mode cracks creates a complex fracture topology, itself a result of stress concentrations around the well bottom.

Figure 2 shows images of the PMMA samples after the hydraulic fracturing tests following procedure P1 (see Methods). All of the samples show two main features: (i) a single mode-I planar fracture that propagated along a vertical plane perpendicular to the direction of the minimum applied stress component and (ii) a set of rotating cracks with complex geometries and originating at different orientations. Hydraulic fractures are expected to propagate along a direction perpendicular to the one of the minimum principal stress component: fractures oriented in such a way are referred to as as critically oriented. However, stress concentrations introduce a non-uniform stress state and, if the deviatoric stress exceeds locally the correspective strenght, a new fracture can initiate at a non-critical orientation: such a fracture will propagate initially perpendicularly to the opening wall, while it will re-aligns perpendicular with the minimum principal stress further away from the perturbed area. At $20\,^{\circ }$C, several minor fractures and cracks are observable around the well at orientations that are non-critical. At higher temperature ($60\,^{\circ }$C), the mixed-mode fractures that propagated from non-critical orientations are more evident and have a larger prevalence than at $20\,^{\circ }$C. The non-critically oriented cracks develop initially along a direction that is perpendicular to the maximum horizontal stress $\sigma _\text{H}$ and rotate during further propagation inside the sample in order to re-align with the direction of the minimum horizontal stress $\sigma _\text{h}$. In all samples, complex three-dimensional fracture patterns are observed, especially at the bottom-end of the well where higher stress concentrations are expected to occur.

**Figure 3:**
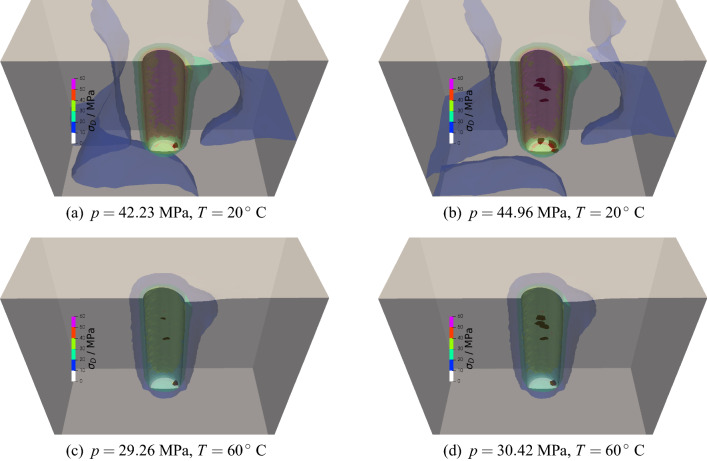
The numerical simulations of the stress concentration effects in procedure P1 (see Methods). The red spots indicate damaged areas ($d\ge 10^{-4}$) and the contour map the deviatoric stress $\sigma _\text{D}$ distribution at the mixed-mode initiation pressure (**a**,**c**) and mode-I propagation pressure (**b**,**d**).

The FEM analyses of procedure P1 (see “Methods” section for additional detail) confirm that the complex crack patterns observed are likely a consequence of the a-symmetric stress concentrations that occur at the well bottom (Fig. 3). The a-symmetric o-ring placed on top of the sample generates an uneven stress distribution in the well direction: inside the o-ring, the stress is identical to the injection pressure, while outside the o-ring, it equals $\sigma _\text{ v}$. The red spots in Fig. 3 represent damaged areas and the contour color map the deviatoric stress $\sigma _\text{D}$ within the deformed samples. At $20\,^{\circ }$C, damage initiates at a non-critical orientation at the bottom of the well when the fluid pressure is $\approx 42$ MPa (Fig. 3a). The deviatoric stress is higher on the side of the o-ring and corresponds to the direction of the mixed-mode crack propagation. According to the plastic-damage constitutive model employed (see Methods), a higher deviatoric stress implies conditions closer to the yield surface and, therefore, to failure. At increasing pressurization, mode-I cracks perpendicular to $\sigma _\text{h}$ propagates when the fluid pressure reaches $\approx 45$ MPa (Fig. 3b): in our model, mixed-mode cracks propagate at a lower pressure than mode-I cracks. The same observation holds valid at $60\,^{\circ }$C, with the exception that the mixed-mode propagation pressure is $\approx 29$ MPa (Fig. 3c) and the mode-I propagation pressure is $\approx 30$ MPa (Fig. 3d). The closer values of the two pressures (onset and propagation) are likely to be related to a smaller deviatoric stress concentration, itself a consequence of the higher deformability of PMMA at increasing temperature.

**Figure 4:**
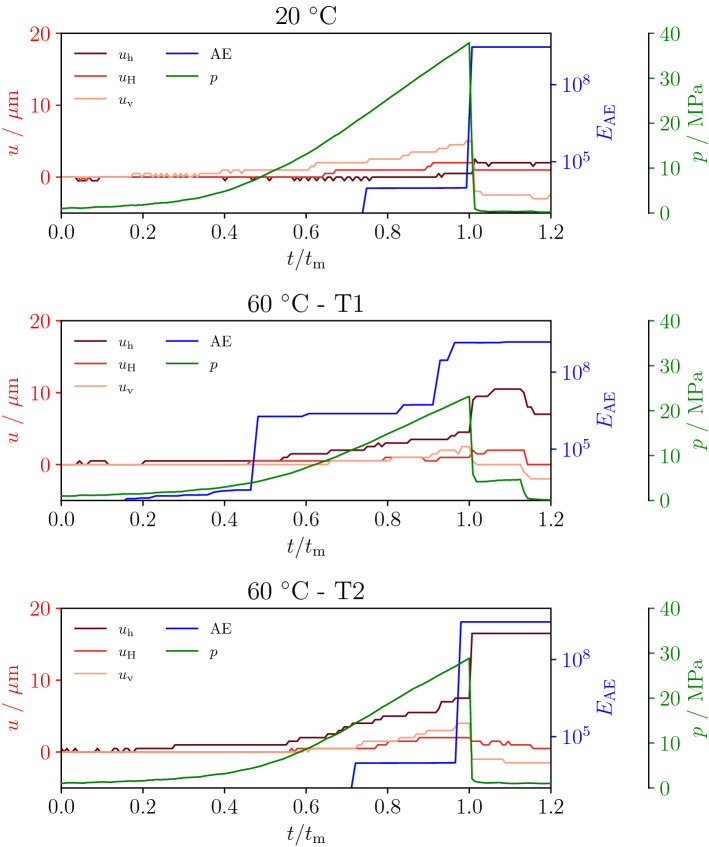
Deformation at the outer faces along the principal directions ($u_\text{h}$, $u_\text{H}$ and $u_\text{v}$) and acoustic emissions’ cumulative energy ($E_\text{AE}$) during fluid injection in test procedure P1 (see “Methods”). Before failure ($t=t_\text{m}$), the onset of AE energy and displacement hints at fracturing process, while the displacements observed along different directions suggests mixed-mode fracture propagation and pre-failure plastic processes.

Figure 4 shows the evolution with time of the three components of displacement recorded at the faces, the cumulative acoustic emissions energy $E_\text{AE}$ and the injection pressure *p*. The displacements are measured at the outer faces of the sample along the minimum $u_\text{h}$, intermediate $u_\text{H}$ and maximum $u_\text{v}$ principal stress directions. The time is normalized as $t/t_\text{m}$, where $t_\text{m}$ is the time at which the sample fails and the injection pressure drops to zero. The normalization is employed for a direct comparison of the time evolution processes leading to failure. In all cases, displacement increases (sample expansion) during the pressurization phase until the propagation of a hydraulic fracture, where the pressure reaches the maximum before the drop. In general, AE are indicative of failure processes and micro-cracking^25^. Close to failure time $t_\text{m}$, the AE cumulative energy also exhibits a sudden jump of several orders of magnitude: this is associated with the sudden release of accumulated elastic energy when the sample fails.

At $20\,^{\circ }$C, the largest displacement component is along the maximum principal stress direction $u_\text{v}$ (Fig. 4) and the AE energy is low until a sudden jump of $\sim 5$ orders of magnitude at sample failure, which corresponds to a reverse in the displacement that is likely connected to stress release after cracking. For both samples at $60\,^{\circ }$C, the largest displacement is $u_\text{h}$ and the overall displacement is larger than the colder test. Additionally, the first (T1) of the two tests performed under the same conditions (T1 and T2) shows a small increase in AE energy slightly before the observed increment in displacement. At the failure, the displacement $u_\text{v}$ along the maximum principal stresses increases together with the AE energy. In both cases, the observations point toward plastic-deformation and micro-cracking coalescence that leads to a mixed-mode rotating fractures propagating from the well in the non-critically oriented directions: the propagation of cracks occurs along several directions around the well and later rotate to a direction perpendicular to $\sigma _\text{h}$. The progressive accumulation of displacement likely indicates that inelastic processes such as micro-cracking and crazing that are taking place earlier during the injection are connected with secondary cracks developing around the well. During test T2, the micro-cracking coalescence is less pronounced, as indicated by a later onset of AE energy and a larger displacement jump toward failure. The recorded data of AE and displacement are consistent with the complex topology of cracks observed in post-mortem samples (Fig. 2).

### Effects of initial stress heterogeneities

**Figure 5:**
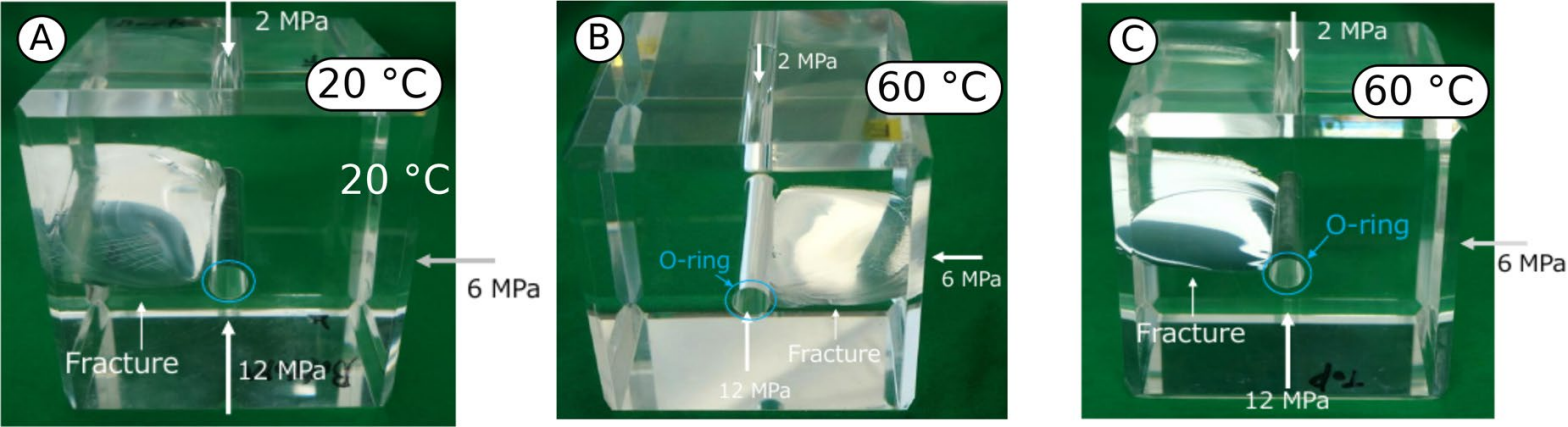
Images of the post-mortem samples tested following procedure P2 (see Methods) at $20\,^{\circ }$C and $60\,^{\circ }$C. The complex fracture pattern observed with procedure P1 disappears, and overall a classical pure mode-I planar hydro-fracture develops independently of temperature.

Procedure P2 (see Methods for a full description) is designed to minimize the deviatoric stress concentrations around the well before fluid injection takes place. The fracture propagation is planar and perpendicular to the minimum principal stress $\sigma _\text{h}$ up to the outer sample wall (Fig. 5). No rotating cracks or complex fracture topology are observed in the post-mortem samples and the behaviour follows a classical mode-I planar hydraulic-fracture propagation.

### Comparison of experimental procedures

**Figure 6:**
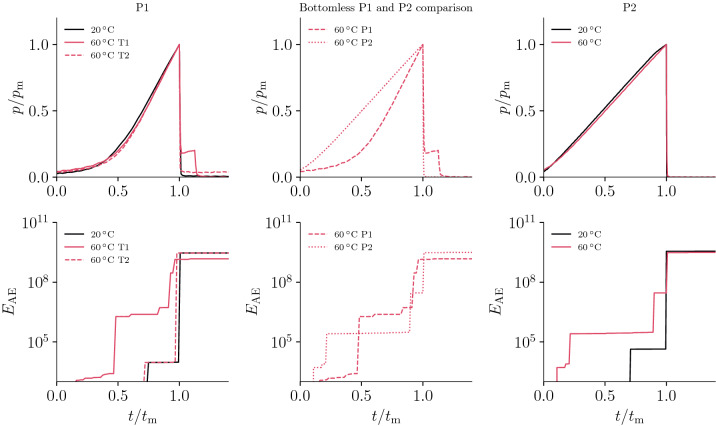
Normalized pressure $p/p_\text{m}$ and cumulative acoustic emission energy $E_\text{AE}$ for all tests carried out in the glassy regime of PMMA. The normalized pressure-time curves are self similar, while the AE energy signature proves that higher temperature and a loading path that promotes deviatoric stress concentration can result in earlier onset of inelastic processes during injection.

We have compared procedures P1 and P2 in terms of pressure and AE cumulative energy (Fig. 6) to highlight the effects of stress concentrations and inelastic processes preceding final failure. For a direct comparison of all tests, the fluid pressure is normalized $p/p_\text{m}$, where $p_\text{m}$ is the maximum pressure reached during testing and corresponds to breakout conditions. All the normalized pressure-time curves are self-similar because of the constant injection rate, and exhibit a linear pressurization phase until the maximum (breakout) pressure is reached and followed by the fracture propagation phase in which the pressure drops to zero.

The acoustic emissions cumulative energy shows an evolution that depends on the temperature and testing procedure adopted. All tests show a similar trend in terms of onset of inelastic processes: the higher the temperature, the earlier the time at which $E_\text{AE}$ has the first increase. For example, for P2 at $20\,^{\circ }$C, the largest part of inelastic energy is suddenly released at fracture failure.

The comparison between procedure P2 and procedure P1, both carried out in a well that extends all throughout the sample, shows that energy dissipation through micro-cracking occurs at earlier stages in procedure P1. The behavior is a consequence of the higher deviatoric stress in P1 (loading sequence): failure and crazing can happen at a lower level of pressure, as evidence by the earlier onset of $E_\text{AE}$.

### Temperature effects in hydro-fracture propagation across the glass transition

**Figure 7:**
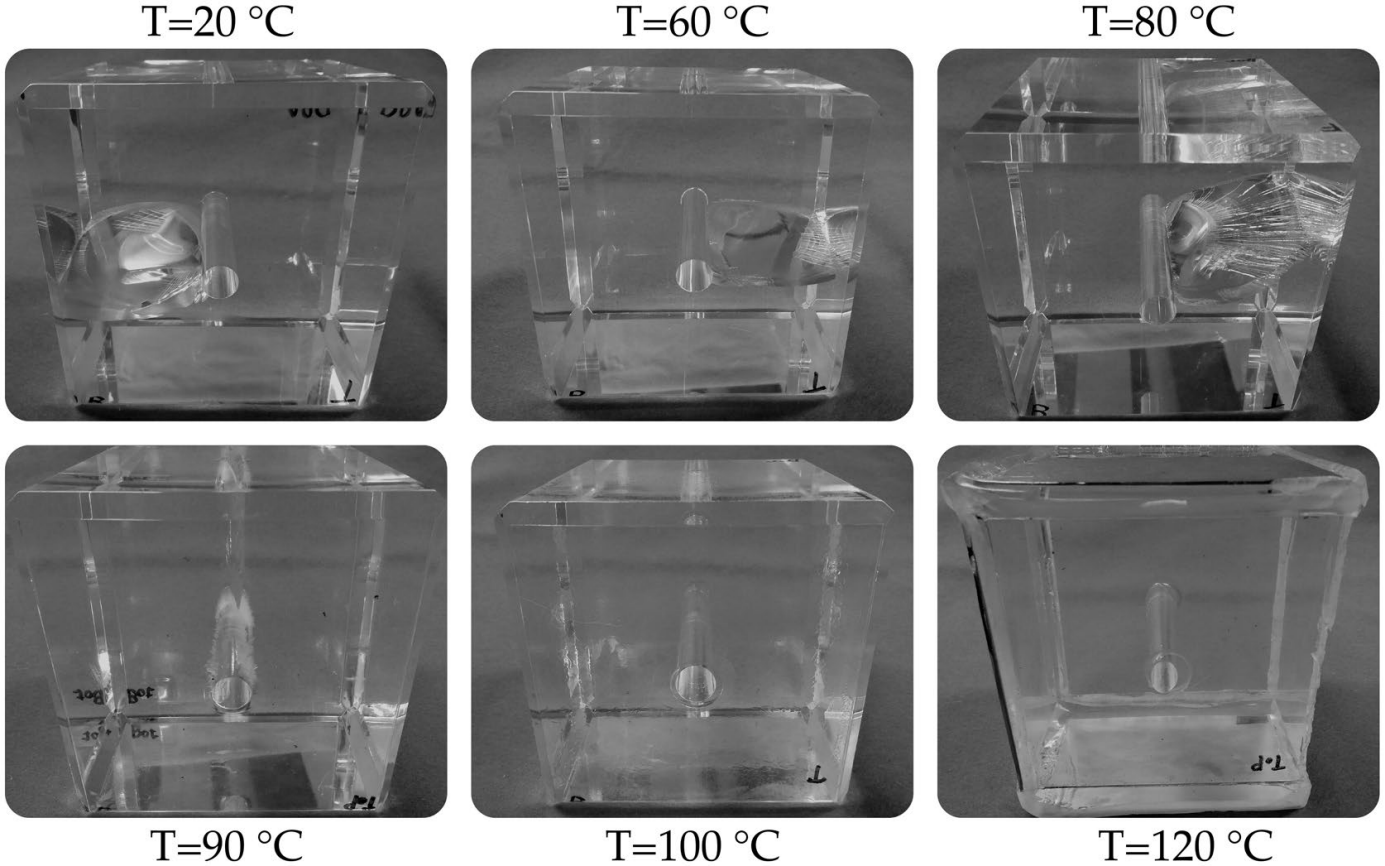
Post-mortem samples of PMMA at different temperatures following P2 experimental procedure (see Methods). A single planar mode-I hydro-fracture propagated up to $T\le 90\,^{\circ }$C, while no fracture occurred at $T\ge 100\,^{\circ }$C where only large visco-plastic deformation around the wellbore was observed.

The full series of experiments following procedure P2 was conducted to study the propagation of planar and mode-I hydraulic fractures in a temperature range $20<T<120\,^{\circ }$C, i.e., fully across the glass transition temperature $0.78<{\tilde{T}}<1.04$. The post-mortem samples show that a single fracture propagated up to $T=90\,^{\circ }$C, while the two tests at $T=100\,^{\circ }$C and $T=120\,^{\circ }$C show signs of large plastic deformation around the well with no fracture propagation (Fig. 7). At $T=90\,^{\circ }$C, crazing was additionally observed close to the wellbore walls, which indicates an increase of micro-cracking and plastic strains with temperature. Close to the glass transition temperature $T_\text{g}$, the hydro-fracturing is hampered. At $T=120\,^{\circ }$C, the large thermally activated visco-plastic deformation caused the sample to extrude from the triaxial apparatus.

**Figure 8:**
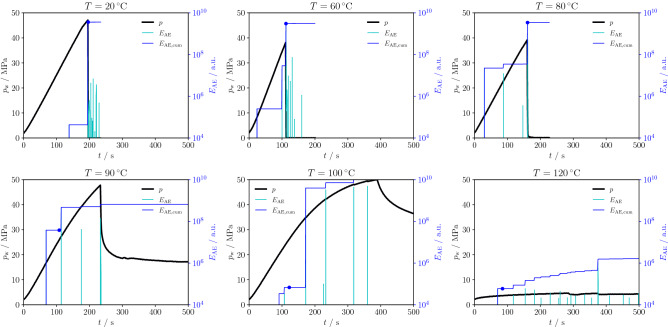
Fluid pressure, acoustic emissions’ energy and cumulative acoustic emissions’ energy recorded during the injection tests at different temperatures and following procedure P2. The pressure response shows a gradual increase in ductility with a slower post-peak drop, while a full transition to practically constant pressure is observed above the glass transition point. Cumulative AE energy at the fracture initiation (blue dot) also gradually decreases with increasing temperature.

The process of hydro-fracturing is affected by the increase in ductility in the PMMA samples with temperature. This feature is visible in the pressure-time curves (Fig. 8) during injection. An elastic-brittle response is observed up to $T=80\,^{\circ }$C, while for $T\ge 90\,^{\circ }$C the fluid pressure shows first a non-linear response characteristic of plastic hydro-fracturing, an a post-peak behaviour that becomes gradually more ductile with a less pronounced pressure drop. The deviation from linearity of the pressure-time curve (see Methods for its detection) is gradually anticipated at earlier time, which corresponds to a decrease of the fracture initiation pressure (non-linear inelastic onset). The cumulative acoustic emission energy corresponding to the fracture initiation onset (non-linear response) decreases with increasing temperature. The two tests at $T\ge 100\,^{\circ }$C have shown no hydraulic fracture propagation and the response is fully consistent with a plastic deformation of the wellbore. Above the glass transition (i.e., $T=120\,^{\circ }$C), we observe a constant strain hardening effect without any stress drop that is instead observed in the low-temperature brittle fracturing (Fig. 8). Since no fracture occurs in the fully ductile regime, the well is continuously pressurized by the injected fluid and the deformation increment in the outer faces of the sample is practically isotropic. The value of $E_\text{AE}$ at the fracture onset is several orders of magnitude smaller than the one observed in the glassy regime—a direct consequence of the transition towards plastic dominated deformation without fracturing.

**Figure 9:**
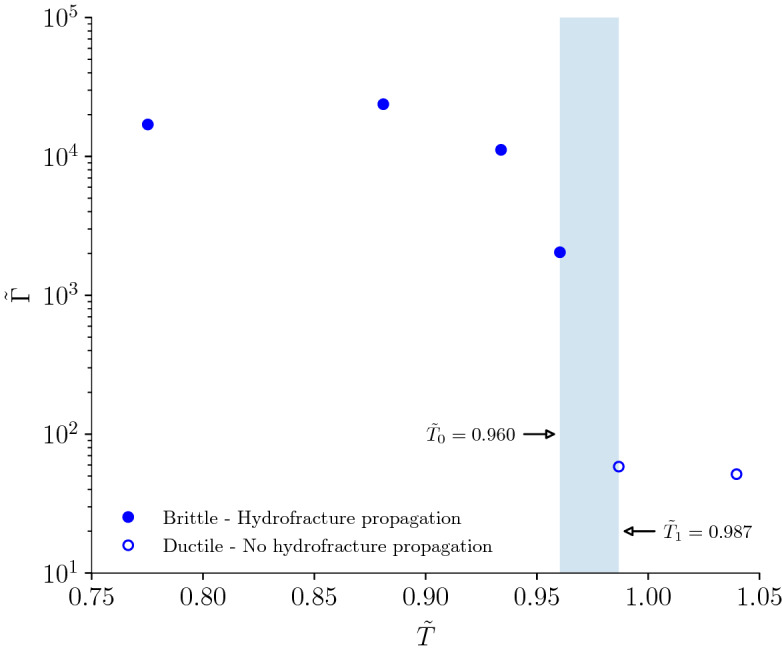
The normalized cumulative acoustic emission energy ${\tilde{\Gamma }}$ is a function of the normalized temperature ${\tilde{T}}$: ${\tilde{\Gamma }}$ decreases with increasing temperature and marks a stark transition of the hydro-fracturing behaviour at the glass transition temperature $T_\text{g}=105\,^{\circ }$C. While fracturing is observed up to ${\tilde{T}}\le 0.96$, no fracture is propagated at ${\tilde{T}}\ge 0.987$.

The normalized cumulative acoustic emission energy ${\tilde{\Gamma }}$ (see Methods for its definition and derivation) is a function of the normalized temperature ${\tilde{T}}$ (Fig. 9). ${\tilde{\Gamma }}$ is approximately constant up to $T=80\,^{\circ }$C and decreases once plastic processes become non-negligible at $T=90\,^{\circ }$C. A sharp decrease of 1 order of magnitude of ${\tilde{\Gamma }}$ occurs in proximity of the glass transition temperature. Once plastic deformation is the dominant mechanism and no hydro-fracture propagates, ${\tilde{\Gamma }}$ is approximately constant. The transition between the regime in which fracture propagates and the one in which no fracture propagates is observed in the interval $0.960\le {\tilde{T}}\le 0.987$. The fully ductile behaviour above the glass transition temperature places the PMMA rheology within the rubbery regime. The large deformations and the low acoustic emissions are a consequence of the strong decrease in stiffness and strength. The lower stiffness and plastic flow mechanisms prevent the formation of micro-cracks and fractures only above $T_\text{g}$ and the inelastic dissipation is essentially aseismic.

## Discussion

Defects and stress concentrations create the conditions for hydraulic fractures propagating non-perpendicular to the minimum principal stress. As the hydro-fractures propagate away from the well, they rotate and propagate in mixed-mode conditions. Stress concentrations at the bottom of a finite-length well^14^ and anisotropy or uneven distribution of strength can also contribute to the initiation and propagation of rotating and non-planar hydraulic fractures^26^. Mixed-mode propagation could be beneficial as mode-II shear is normally associated with dilatancy and permanent opening of fractures in a reservoir that contribute to the permanent permeability increase. Mix-mode fracturing is affected by the shear failure behaviour of rocks, which is itself strongly influenced by the changes that occur across the brittle-ductile transition. Therefore, the brittle-ductile transition can be expected to have a particularly relevant influence whenever heterogeneous conditions will promote the propagation of non-planar and rotating hydraulic fractures.

Our results demonstrate that the upper limit to the propagation of hydraulic fractures is close to the glass transition temperature $T_\text{g}$. The limit is strictly connected with the rheological behaviour of PMMA and an increase in ductility does not necessarily translates into a non-fracturing behaviour. We have shown that below the glass transition temperature, for example at $90\,^{\circ }$C, planar and pure mode-I fracturing still occurs, despite the increased plastic deformation and ductility^20^. Uniaxial tensile tests from literature have shown that up to $T/T_\text{g}\sim 0.93$, PMMA undergoes a strain-softening phase followed by re-hardening up to breakage and failure^21^. Final failure and breakage ultimately disappears approximately at $T/T_\text{g}\sim 0.97$, i.e., when the rheology is about to enter the rubbery regime and fully ductile deformation takes place. Our findings are consistent with the previously observed rheological behavior of PMMA.

From the rheological observations, we show that hydro-fracturing attenuation occurs only once the material can accommodate very large values of plastic deformation without ultimate failure. Such a limit in igneous (even in certain metamorphic rocks) is believed to lie well above the limit of $600\,^{\circ }$C^27–29^. Evidence suggests that at $450\,^{\circ }$C, hydro fracturing occurs through fluid percolation: micro-cracks and an increase in permeability have been observed in post-mortem samples^5^. Natural seismicity is known to occur well above $500\,^{\circ }$C and possibly even $T>600\,^{\circ }$C^27^, and brittle-ductile transition models have shown that the presence of fractures and faults is almost certain in such high temperature conditions^29^. Furthermore, drilling of the IDDP-2 well has generated induced seismic events at temperatures above $550\,^{\circ }$C^6^ and the high permeability recorded is likely associated with permeable fractures.

Glass content in rocks affects the transition between brittle to ductile deformation mode in confined shear: experiments have shown that the transition temperature in glassy basalt can drop from $\ge 700\,^{\circ }$C in glass-free samples to $500\,^{\circ }$C in samples containing approximately 20% glass^28^. In steady-state creep deformation, glassy basalt further exhibits a large strength drop occurring most likely across the glass transition temperature^30^, where the latter is influenced by water and crystal content and by the oxygen fugacity in silicate glasses^31^. Dikes propagate in the crust through mechanisms that share several similar features to hydraulic fractures^32^ and the rheology of the rock is likely to influence the mechanisms of propagation. Close to the glass transition temperature, the ductility of the host rock could hamper the propagation of magma through fracturing; in other words, the increased ductility in the hot crust could alter the dike propagation mechanisms when compared to the colder brittle crust.

In high-temperature geothermal systems and for other natural processes in which a pressurized fluid flows in porous and fractured rocks, the rheological features of the solid is coupled with the intrinsic fluid-conductive nature of the rock. Two kinds of fluid-solid interactions are likely to dominate the physics of high-temperature hydraulic fracturing: (i) effective stress changes and (ii) chemically induced processes of dissolution and precipitation.

The first process is connected to the fluid flow through the porous structure of the rock, where it changes its effective stress $\sigma '$ in function of the pore pressure $p_\text{f}$ as $\sigma '=\sigma -\alpha p_\text{f}$, where $\alpha$ is Biot’s coefficient and $\sigma$ the total stress. Below the critical temperature, multi-phase flow could occur and capillary forces on the solid skeleton arising at the phase contact generate an effective stress $\sigma '=\sigma -p_\text{g}+\chi \left( p_\text{g}-p_\text{f}\right)$^33^, where $p_\text{g}$ is the gas pressure and $\chi$ is Bishop’s parameter, with a common choice of $\chi =S$^34^, where *S* is the liquid saturation. Although several other expressions have been proposed in literature^35^, the role of capillary forces in water-vapour geothermal systems is usually neglected due to the scarcity of evidence in the high-temperature range^36^ and the decrease in surface tension with temperature. Whichever the framework, changes in the liquid and/or gas pressure lead to changes in the effective stress and possible tensile or shearing failure induced micro-cracking, which has been observed in high-temperature hydro-fracturing experiments or rocks^5,9^. Furthermore, the global wetting-phase permeability decreases with the increase of the non-wetting-phase saturation, affecting the fluids pressure distribution in multi-phase fractured rocks^37,38^, which in turn affects the effective stress distribution acting on the solid skeleton.

The second processes is related to geochemical alterations and reactions of dissolution and precipitation of minerals, such as silica, as fluid flows through the pores and fractures network. At relatively low temperature, as water flows through fractures, the pressure-solution reactions increase the contact area and reduce the aperture, generating a creep-like effect in fracture permeability^39^. Close to the fluid’s critical point, quartz solubility in water increases with temperature before dropping to low values at temperatures above the critical point, where it could generate deposition-clogging of the porous structure on a medium to long-term scale^40^. On the other hand, free face dissolution overcomes the reduction of fracture aperture if the difference between the quartz concentration in the pore water and its solubility is kept above a specific threshold^41^.

Our observations pose an additional challenge to the widespread assumption that makes supercritical geothermal systems coincide with the ductile crust: field observations on natural and drilling-induced seismicity, rheological laboratory evidence, flow-through fractures experiments and our hydro-fracturing experiments on rock analogues all provide important evidence that points toward a brittle crust in igneous provinces that likely extends up to $\approx 600\,^{\circ }$C. Hydraulic fracturing is likely to be successful when applied to stimulate supercritical geothermal reservoirs in the range $374\,^{\circ }$C$<T<600\,^{\circ }$C; however, this limit is yet to be further investigated by collecting additional evidence from laboratory experiments as well as from field-scale applications. Several factors are expected to influence the propagation of hydraulic fractures and the permeability enhancement in supercritical geothermal reservoirs, such as the fluid equation of state and its influence on flow conditions and fracture-to-matrix leak-off, the role of the physical and micro-mechanical processes controlling rock deformation and micro-fracturing mechanisms and the fluid-solid dissolution/precipitation interactions. Furthermore, as the solid deformation mechanisms strongly depend on rock lithology, different formations are expected to exhibit rather different thresholds of hydro-fracture propagation. Additionally, further tests on real rock samples are necessary as some of the characteristics of shear-failure in rock-like materials can hardly be reproduced in PMMA^42^. Although our physical inferences are based on rheological analogies and not on direct measurements on rock samples, future studies can strongly benefit from our findings: the strength, ductility and thermally activated plastic and reactive processes of rocks are expected to play a fundamental role in hydro-fracturing at $T\ge 400\,^{\circ }$C.

## Conclusions

We have studied the propagation of hydraulic fractures in PMMA at temperatures spanning the rheological extremes of brittle and ductile deformation mode. We have further investigated the effect of stress concentrations and a-symmetric stress states in the development of complex crack patterns.

Our results show that temperature has an important effect on the hydraulic fracture propagation in PMMA and that the glass transition temperature $T_\text{g}$ is the most important parameter. Close to the glass transition temperature, no hydraulic fracture occurs; below that limit, the propagation mode of hydraulic fractures is controlled by stress concentrations and temperature dependent inelastic processes prior to final failure. In brittle conditions, inelastic processes and stress concentrations can lead to complex fracture topology and mixed-mode rotating crack propagation.

Because of all of the complexities related to the rheology of the rock, the role of the fluids and their mutual interaction, the extrapolation of our results on PMMA toward geothermal systems still bears uncertainties that can be removed only by further investigations on actual rocks. Nonetheless, we have clearly demonstrated that (i) when the solid rheology is semi-ductile, hydro-fracturing still occurs and (ii) when the solid rheology is purely ductile, it is nearly impossible to propagate hydraulic fractures. What is still to be discovered, is whether these conclusions will remain valid for rocks in the ductile or semi-ductile spectrum and when the fracturing process is fluid percolation induced micro-cracking.

Finally, we can conclude that supercritical geothermal systems intersect a part of the crust that behaves in a brittle way and in which hydro-fracturing is likely to be successful: that section of the upper crust is defined by a low bound that coincides with the critical point of water ($\approx 374\,^{\circ }$C) and an upper bound that coincides with the likely limit of brittle deformation in rock ($\approx 600\,^{\circ }$C).

## Methods

### True-triaxial apparatus

**Figure 10:**
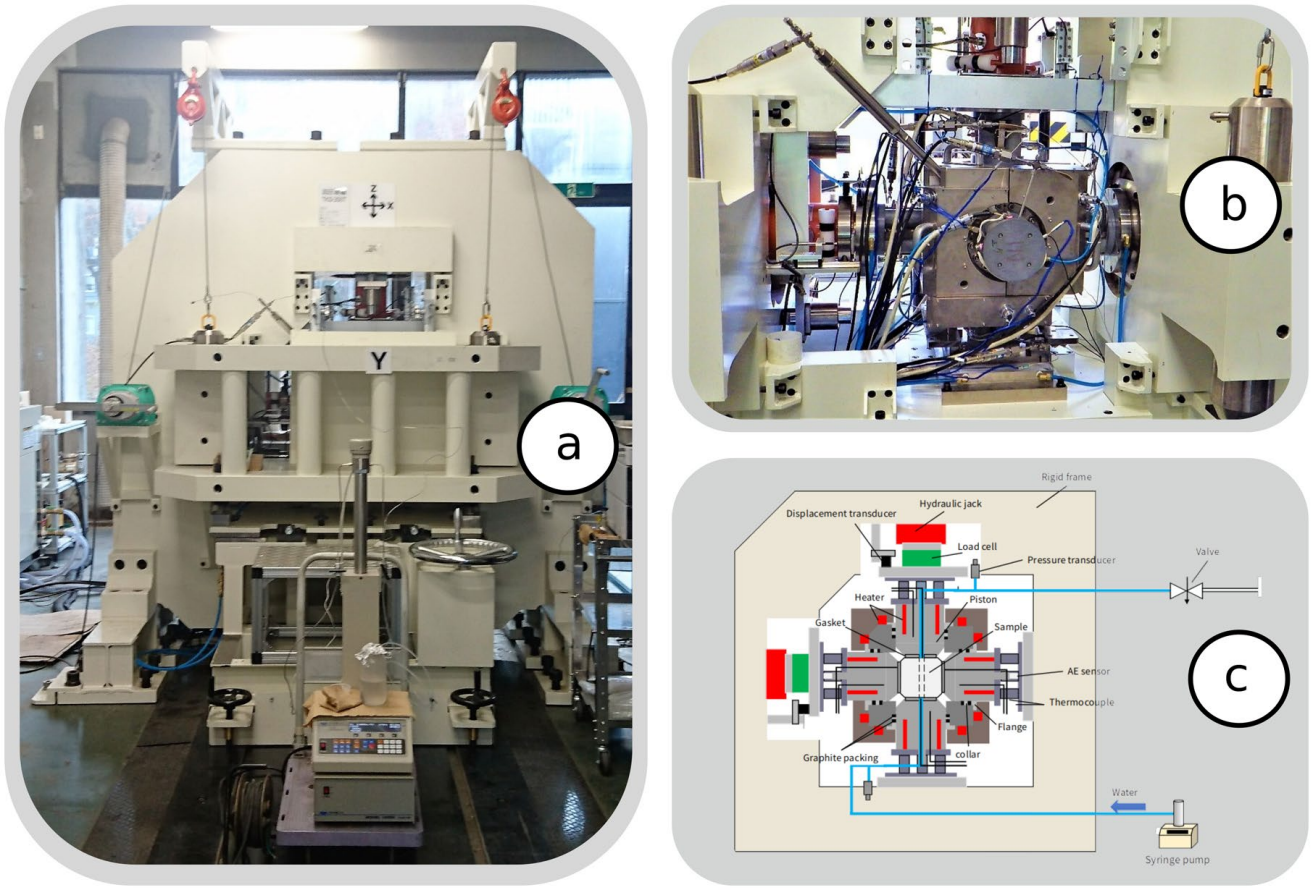
The experimental apparatus is composed of a loading frame (**a**), a true triaxial cell (**b**) and a system of pumps and measuring devices to conduct the experiments at temperatures up to $500\,^{\circ }$C (**c**).

The experimental system consists of a triaxial loading system (Fig. 10a), a true triaxial cell (Fig. 10b), pumps (syringe pump or plunger pump) for injecting fluids, an elastic wave measurement system (P-wave or acoustic emission measurement system), and a temperature control and data logging system (all shown in the detailed Fig. 10c). The elastic wave measurement system in the present study is an acoustic emission (AE) measurement device (Physical Acoustics Cooperation’s two-channel data acquisition and digital signal processing AE system, PCI-2). The cell consists of a pressure vessel with a cubic skeleton, six pistons that apply a compressive load to the sample via a stainless-steel gasket, and thermal insulators used in conjunction with heaters for the pressure vessel. The pressure vessel has six cylindrical holes to allow the pistons to be inserted into the vessel, with graphite packing placed at the sliding portion. The piston has a square loading face (90 mm $\times$ 90 mm) and an elastic wave guide bar at the opposite side of the loading face. An AE sensor (R15$\alpha$ 150-kHz resonant frequency sensor, Physical Acoustics Corporation) is attached to the end face of the elastic wave guide bar, and two AE sensors are used for a pair of opposed horizontal pistons. The AE energy is reported in arbitrary units (a.u.), where the fundamental reference unit corresponds to $1\,\mathrm {a.u.}=9.31\times 10^{-22}$ J.

In addition, the temperature of the bar can be maintained near ambient conditions using a cooling jacket through which water from a chiller circulates. The piston is equipped with a pipe that acts as a flow path for injecting or producing fracturing fluid, and four cartridge heaters with a thermocouple inside the pipe to measure the temperature near the sample face. The pipe can be connected to a pump, a pressure transducer, or a valve depending on the purpose of the experiment.

### Experimental procedure

**Figure 11:**
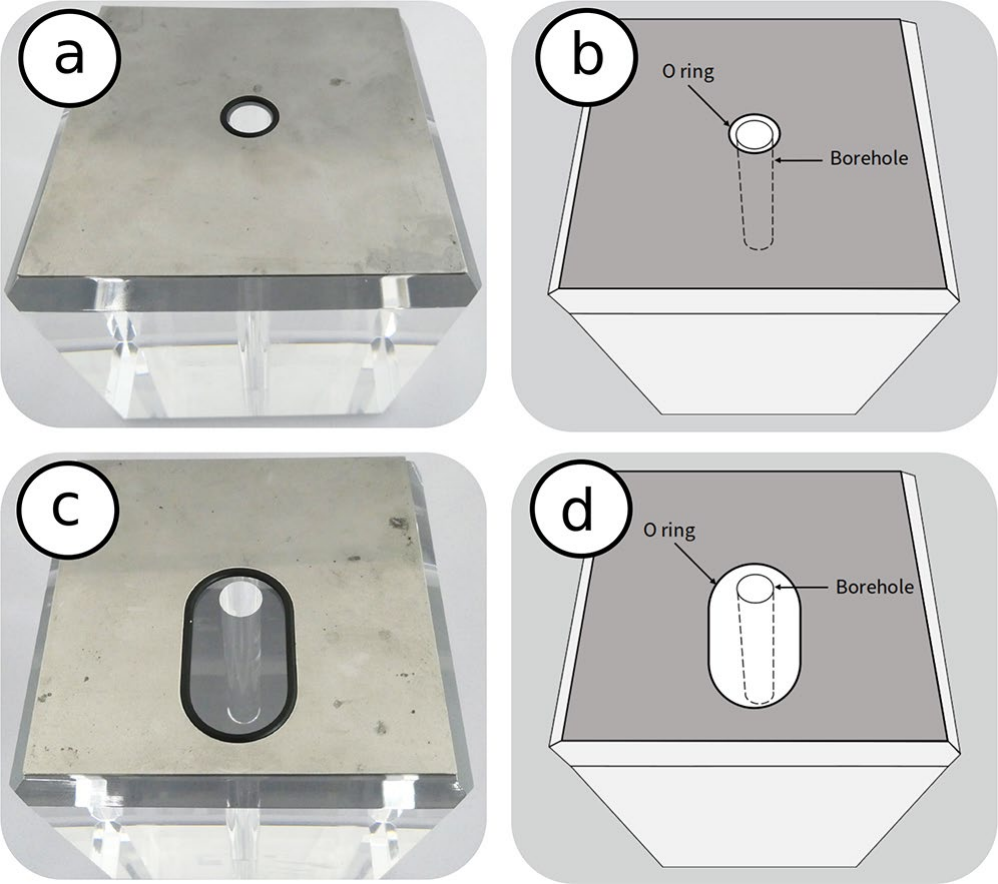
Chamfered cubic samples of PMMA with a circular (**a**,**b**) or an elliptical (**c**,**d**) opening of the stainless steel gasket. The elliptical configuration is employed to generate a non-uniform and non-symmetric stress distribution in the sample.

Cubes (100 mm $\times$ 100 mm $\times$ 100 mm) of polymethyl methacrylate are prepared from methyl methacrylate (MMA), produced by Kuraray Co., Ltd., with a single vertical borehole (diameter: 10 mm, length: 50 mm or 100 mm) at the center for the hydraulic fracturing experiments under true triaxial stress (Fig. 11). The edges of the cubic PMMA sample are chamfered so that the loading face of the sample has 90 mm sides to be adapted to the hydraulic fracturing experiment system^5^.

We have designed two separate procedures in an attempt to highlight the effects of stress concentrations and micro-cracking phenomena around the well and their influence in mixed-mode hydraulic fracture propagation. In all tests the applied true triaxial stress corresponds to $\sigma _\text{v}>\sigma _\text{H}>\sigma _\text{h}$, with $\sigma _\text{v}$ is applied along the direction of the well, and $\sigma _\text{H}$ and $\sigma _\text{h}$ are the maximum and minimum horizontal stresses, respectively. The minimum, intermediate and maximum principal stresses are, respectively, $\sigma _3=\sigma _\text{h}$, $\sigma _2=\sigma _\text{H}$ and $\sigma _1=\sigma _\text{v}$. The stainless-steel gasket have a circular (Fig. 11a,b) or an elliptical (Fig. 11c,d) hole with o-ring that is placed between the piston for fluid injection and the loading face of the sample. The stainless-steel gasket without holes is placed between the other piston and the loading face of the sample. The use of the gasket with the elliptical hole results in asymmetric o-ring setting around the borehole, whereas the use of the gasket having the circular hole results in a symmetric o-ring setting.

**Figure 12:**
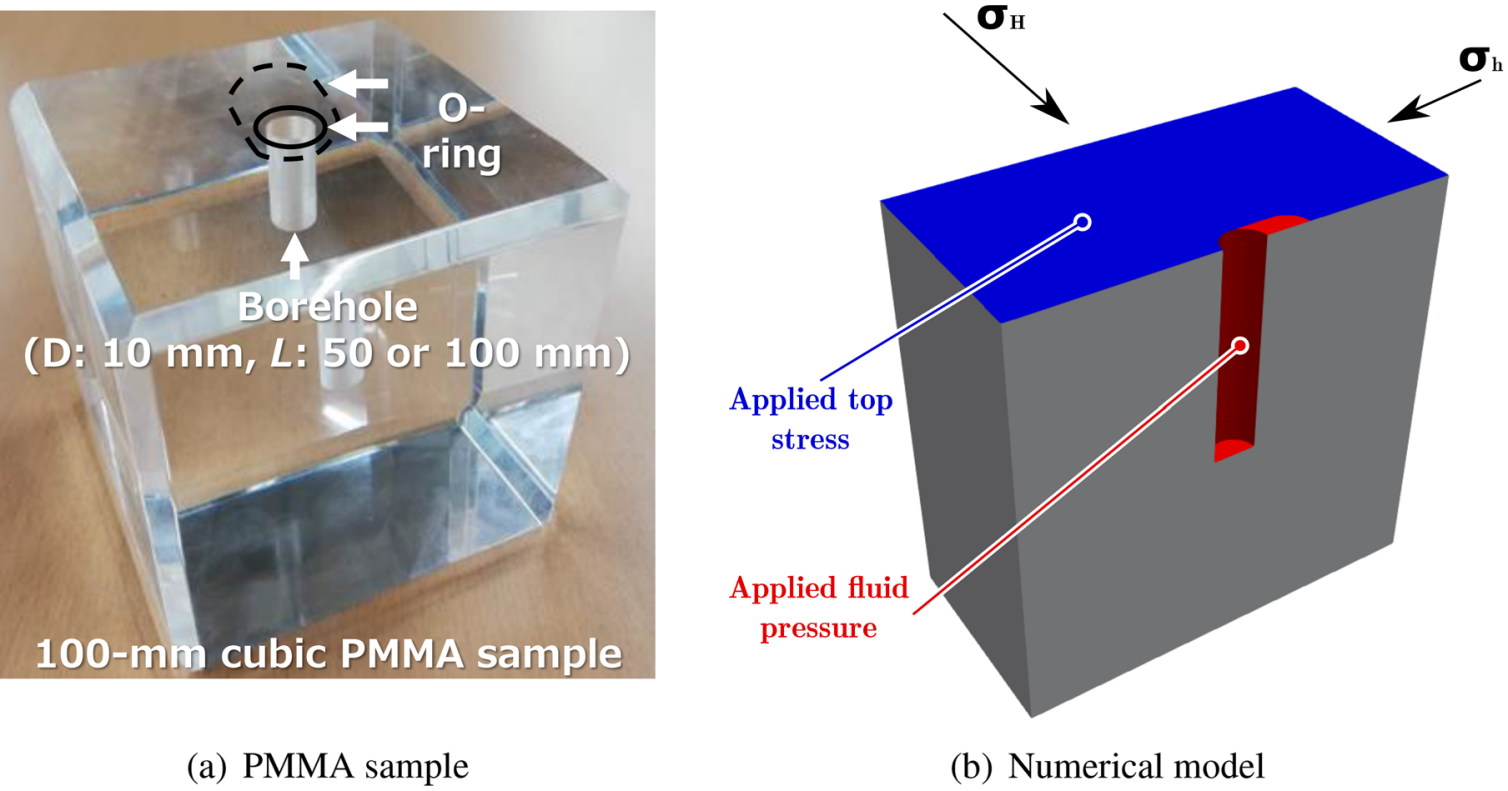
Sample of PMMA (**a**) and configuration of test P1 (**b**) with a bottom well and an a-symmetric o-ring that generates an additional stress concentration on one side of the well.

The first experimental procedure (P1) is carried out on a sample with a borehole that does not cross the whole sample but instead penetrates up to the middle of the sample (bottom sample) and is designed to highlight the mixed-mode propagation by generating stress concentrations around the opening (Fig. 12a). Additionally, a non-symmetric o-ring is placed at the top injection side, such that a non-homogenous pressure will be applied on the top surface and in a direction parallel to the well (Figs. 11c,d and 12b). The tests are carried out at temperatures $T_\text{f}=20$ $^{\circ }$C and $T_\text{f}=60$ $^{\circ }$C. The stress path procedure is further designed to maximize the deviatoric stress around the well and is the following:

1. Increase *T* to $$T_\text{f}$$;
2. Increase simultaneously $\sigma _\text{v}$ to 12 MPa (15 MPa at $T_\text{f}=20$ $^{\circ }$C), $\sigma _\text{H}$ to 6 MPa and $\sigma _\text{h}$ to 2 MPa;
3. Inject fluid into the well at a constant rate $q_0=1$ ml $\hbox {min}^{-1}$.

The asymmetric o-ring is placed at $90^{\circ }$ orientation with respect to the fracture initiation angle (Fig. 12). In pure mode-I, the fracture is expected to propagate perpendicular to the minimum stress $\sigma _\text{h}$.

The second experimental procedure (P2) is designed to minimize the deviatoric stress concentrations before fluid injection, is performed on a well that crosses the whole sample (bottomless), has a symmetric o-ring (uniform stress) and is carried out in the following way:

1. Increase *T* to $$T_\text{f}$$;
2. Increase *p* to 2 MPa;
3. Increase $\sigma _\text{v}=\sigma _\text{H}=\sigma _\text{h}$ to 2 MPa;
4. Increase $\sigma _\text{v}=\sigma _\text{H}$ to 6 MPa ($\sigma _\text{v}=\sigma _\text{H}=4$ MPa at $T_\text{f}=120$ $^{\circ }$C);
5. Increase $\sigma _\text{v}$ to 12 MPa ($\sigma _\text{v}=4$ MPa at $T_\text{f}=120$ $^{\circ }$C);
6. Inject fluid into the well at a constant rate $q_0=1$ ml $\hbox {min}^{-1}$.

The tests are performed at $T_\text{f}=20$ $^{\circ }$C, $T_\text{f}=60$ $^{\circ }$C, $T_\text{f}=80$ $^{\circ }$C, $T_\text{f}=90$ $^{\circ }$C, $T_\text{f}=100$ $^{\circ }$C and $T_\text{f}=120$ $^{\circ }$C. Because of lower strength and creep deformation, it was not possible to apply a higher confining stress at $T_\text{f}=120$ $^{\circ }$C. Further insights are given by a cross-procedure in which steps of P1 are applied to a sample with a bottomless well and a symmetric o-ring as it is for P2, and at $T_\text{f}=60$ $^{\circ }$C.

### Rheology and material properties of PMMA

Like other amorphous solids such as polymers and glasses, many of the temperature dependent mechanical properties of PMMA are controlled by the glass transition temperature $T_\text{g}$^43^. For PMMA, this temperature is approximately $T_\text{g}=378$ K, although the production process can have an influence and wider ranges are usually reported in literature. The behaviour is classified as a function of temperature in the following ways^21^: (i) at $T<T_\text{g}$, the behavior of PMMA is the one of a brittle to semi-brittle solid and is classified as the glassy regime; (ii) at a temperature $T\sim T_\text{g}$, the mechanical properties greatly change with small temperature increment and the regime is called the glass transition regime; (iii) at $T>T_\text{g}$, the properties of PMMA are increasingly dominated by creep and viscous deformation and the two subsequent regimes are called rubbery (iii-a) and viscous (iii-b) regimes. Ductility increases with temperature and within the glassy regime, crazing, fracturing, yielding and localized shear deformations are observed in the temperature range $20-80\,^{\circ }$C^20^.

#### The glassy regime

The work of^20^ reports the formation of cracks and crazes in PMMA at the lower end temperature, while plastic yielding and localized banding are observed at $T>60\,^{\circ }$C. We employ a linear interpolations of experimental data^20^ to approximate the observed decay of tensile strength $\sigma _\text{t,f}$ (fracture/yield), onset of inelastic strain $\sigma _\text{t,0}$ (crazing/shear band onset) and Young’s modulus *E* with temperature as

1 $$\begin{aligned} \begin{aligned} \sigma _\text{t,f}&=-0.582T+240.7\\ \sigma _\text{t,0}&=-0.4T+167\\ E&=-17.77T+8244, \end{aligned} \end{aligned}$$

with stresses expressed in MPa and temperatures in K. The uniaxial compressive strength is related to the tensile via

2 $$\begin{aligned} \sigma _\text{c}=\sigma _\text{t}\frac{3+\alpha }{3-\alpha }, \end{aligned}$$

which, with $\alpha =0.4$, yields $\sigma _\text{c}\approx 1.3\sigma _\text{t}$^21^. According to Equation 1, the uniaxial tensile strength at room temperature is $\sigma _\text{t}\approx 70$ MPa, such that it fits the value reported by the manufacturer. Poisson’s ratio $\nu =0.35$ is assumed to be constant.

To analyze the effects of stress concentrations around the well, we simulate the pressurization problem in the glassy regime and we approximate the behaviour of PMMA with a plastic-damage constitutive theory^44^

3 $$\begin{aligned} {\varvec{\sigma }}=\left( 1-d\right) {\varvec{{E}}}: \left( \varvec{\varepsilon} -\varvec{\varepsilon}^{{\rm p}} \right), \end{aligned}$$

where *d* is damage, $\varvec{\varepsilon }$ is the total strain tensor, $\varvec{\varepsilon }^\text{ p }$ is the plastic strain tensor and ${\varvec{{E}}}$ is the elastic stiffness tensor. The flow rule of plastic strain writes

4 $$\begin{aligned} \dot{\varvec{\varepsilon }}^\text{ p } = {\dot{\lambda }} \frac{\partial g_\text{ p }}{\partial \tilde{\varvec{\sigma }}} \end{aligned}$$

where ${\dot{\lambda }}$ is the plastic multiplier and $g_\text{ p }$ is the plastic potential function. The plastic failure surface characterizes the strength envelope, $f_\text{ p }$, ($f_\text{ p } =g_\text{ p }$) and is of the Drucker-Prager type

5 $$\begin{aligned} f_\text{ p }=\sqrt{J_2}-\beta I_1+k=0, \end{aligned}$$

and the invariants of the stress tensor are

6 $$\begin{aligned} I_1&=\text {tr}\left( \tilde{\varvec{\sigma }} \right) \nonumber \\ J_2&=\left( \tilde{{\mathbf {s}}} {\mathbf {\,:\,}}\tilde{{\mathbf {s}}} \right) /2, \end{aligned}$$

where $\tilde{{\mathbf {s}}}= \tilde{\varvec{\sigma }} - \frac{1}{3}\text {tr}\left( \tilde{\varvec{\sigma }} \right) {\mathbf {I}}$. The plastic surface parameters can be expressed as functions of the compressive and tensile strength as

7 $$\begin{aligned} \begin{aligned} \beta&=\frac{1}{\sqrt{3}}\left( \frac{\sigma _\text {c}-\sigma _\text {t}}{\sigma _\text {t}+\sigma _\text {c}}\right) \\ k&=\frac{2}{\sqrt{3}}\left( \frac{\sigma _\text {t}\sigma _\text {c}}{\sigma _\text {t}+\sigma _\text {c}}\right) \end{aligned}. \end{aligned}$$

The temperature dependence of strength and stiffness is taken into account in terms of uniaxial tensile and compressive strength and Young’s modulus following Equation 1. Since the ratio $\sigma _\text{c}/\sigma _\text{t}=1.3$ is constant, it follows that $\beta =0.08$ is also constant. Damage increments are a function of non-local plastic strain variable

8 $$\begin{aligned} d =\omega \left( {\bar{k}}_d\right) = \left( 1-\beta _d\right) \left[ 1-\exp \left( -\frac{{\bar{k}}_d}{\alpha _d} \right) \right] , \end{aligned}$$

where $\alpha _d=2.0\times 10^{-3}$ and $\beta _d=1.0\times 10^{-3}$ are damage material parameters and

9 $$\begin{aligned} {\bar{k}}_d\left( {\mathbf {x}} \right) = f\left( k_d\left( {\mathbf {x}} \right) \right) = \int _{V} \alpha \left( {\mathbf {x}},\varvec{\xi } \right) k_d\left( \varvec{\xi } \right) \,\mathrm {d}\varvec{\xi }, \end{aligned}$$

where $\alpha$ is the normalized weight function expressed as

10 $$\begin{aligned} \alpha \left( {\mathbf {x}},\varvec{\xi } \right) =\frac{\alpha _0 \left( \left\Vert {\mathbf {x}}-\varvec{\xi }\right\Vert \right) }{\int _{V} \alpha _0 \left( \left\Vert {\mathbf {x}}-\varvec{\psi }\right\Vert \right) \,\mathrm {d}\varvec{\psi }}, \end{aligned}$$

with the distance measure $r=\left\Vert {\mathbf {x}}-\varvec{\xi }\right\Vert$ and the weight function

11 $$\begin{aligned} \alpha _\text {0}= {\left\{ \begin{array}{ll} \displaystyle \left( 1-\frac{r^2}{l_{\text {c}}^2}\right) &{} \text {if } 0 \le r \le l_\text {c} \\ 0 &{} \text {if } l_\text {c} \le r \\ \end{array}\right. }, \end{aligned}$$

with $l_\text {c}=0.1$ mm is the internal length. Damage depends on the plastic strain measure ${\dot{k}}_d={\dot{\varepsilon }}^\text{ p}_{\text {eff}}$ and

12 $$\begin{aligned} {\varepsilon }^\text{ p}_{\text {eff}}(t)=\int \limits _0^t \sqrt{ \frac{2}{3} {\dot{\varvec{\varepsilon}}}^{\rm p}: \dot{\varvec{\varepsilon}}^{\rm p}}\, {\mathrm{d}}\tau. \end{aligned}$$

We have built a 3D FEM model with the software OpenGeoSys (www.opengeosys.org) to investigate the influence of the non-symmetric deviatoric stress in terms of failure initiation patterns around the well (Fig. 12b).

#### Rheology at the glass transition regime

The strength and stiffness of PMMA decay when the temperature is approaching $T_\text{g}$ and can be modeled according to the model presented by^21^, which we report in the following. The secant Young’s modulus in the glassy regime and in the glass transition regime is

13 $$\begin{aligned} E_\text{S}=\eta \frac{{\dot{\varepsilon }}}{\varepsilon _\text{ref}}\left[ 1-\exp {\left( -\frac{E_\text{g} \varepsilon _\text{ref}}{\eta {\dot{\varepsilon }}}\right) }\right] , \end{aligned}$$

where $\eta$ is a viscosity, ${\dot{\varepsilon }}$ is the loading strain rate, $\varepsilon _\text{ref}=0.05$ is a reference strain and $E_\text{g}$ is the so-called glassy modulus. The viscous deformation is based on the theory of Williams-Landel-Ferry^45^

14 $$\begin{aligned} \eta =3\eta _0\exp {\left[ \frac{-\ln {\left( 10\right) }C_1\left( T-T_\text{g} \right) }{C_2+T-T_\text{g}} \right] }, \end{aligned}$$

with the reference viscosity $\eta _0=6.7\times 10^{8}$ Pa $\hbox {s}^{-1}$ and constants $C_1=42.77$ and $C_2=113.8$ K^21^. The glassy modulus is a function of temperature

15 $$\begin{aligned} E_\text{g}=E_0\left( 1-\alpha _\text{m}\frac{T}{T_\text{g}}\right) , \end{aligned}$$

with the absolute zero modulus $E_0=3.52$ GPa and $\alpha _\text{m}=0.85$^21^. In the rubbery regime of deformation, the empirical relation of Young’s modulus writes

16 $$\begin{aligned} E_\text{R}=E_\text{R0}\left( 1-\alpha _\text{R}\frac{T}{T_\text{g}}\right) \left( \frac{{\dot{\varepsilon }}}{{\dot{\varepsilon }}_\text{R}}\right) ^n, \end{aligned}$$

where the reference modulus $E_\text{R0}=65.8$ MPa and strain rate ${\dot{\varepsilon }}_\text{R}=1.58$ $\hbox {s}^{-1}$, the temperature coefficient $\alpha _\text{R}=0.8$ and the rate sensitivity index $n=0.173$^21^. The overall Young’s modulus *E* around the glass transition regimes is given by

17 $$\begin{aligned} E=\max {\left( E_\text{S},E_\text{R}\right) }. \end{aligned}$$

The uniaxial tensile strength in the glassy regime is

18 $$\begin{aligned} \sigma _\text{t}=\frac{{\bar{\sigma }}}{1+\alpha /3}, \end{aligned}$$

and the failure stress ${\bar{\sigma }}$ is related to the elastic strain rate ${\dot{\varepsilon }}_\text{e}$ via

19 $$\begin{aligned} \frac{{\dot{\varepsilon }}_\text{e}}{{\dot{\varepsilon }}_0}=\sinh {\left( \frac{{\bar{\sigma }} \mathrm {v}}{\mathrm {k}T}\right) }\exp {\left( -\frac{\mathrm {q}}{\mathrm {k}T}\right) }, \end{aligned}$$

where ${\dot{\varepsilon }}_0=1.5\times 10^{56}$ $\hbox {s}^{-1}$ is a reference strain rate, $\mathrm {v}=1.62\times 10^{-27}$ $\hbox {m}^3$ is an activation volume and $\mathrm {k}$ is the Boltzmann constant^21^. Tensile failure in the rubbery regime is given the flow law

20 $$\begin{aligned} \sigma _\text{t}=E_\text{R}\varepsilon _\text{ref}. \end{aligned}$$

The complex configuration with the well at the center makes it difficult to apply a specific strain rate. We have estimated from numerical analyses that the strain rate is approximately ${\dot{\varepsilon }}\approx 3\times 10^{-4}$ $\hbox {s}^{-1}$ in the current experimental conditions. The curves of strength $\sigma _\text{y}$ and stiffness *E* in the transition regime are employed to compare the observed acoustic emissions across the glass transition.

#### Normalized acoustic emission energy

The critical energy release rate $G_\text{c}$ is computed at the peak of stress as

21 $$\begin{aligned} G_\text{c}=g_\text{f}\frac{V}{2A}, \end{aligned}$$

where the dissipated energy per unit volume at peak strength is given by

22 $$\begin{aligned} g_\text{f}=\frac{1}{2}\frac{\sigma _\text{t}^2}{E}, \end{aligned}$$

and the fracture volume and area are, respectively, *V* and *A*. The ratio $V/A=w_\text{f}$ is identical to the width of the dissipation zone perpendicular to the fracture surface. In this case, $w_\text{f}\approx 0.26$ mm is back-calculated by assuming the properties of PMMA from the previous section and a typicial value of critical energy release rate at ambient temperature^46^, i.e., $G_\text{c}\approx 500$ J $\hbox {m}^{-2}$.

The cumulative acoustic emission energy is ${\bar{E}}$ is taken at the onset of the deviation of 1% from linearity in the pressure curve during injection (see Fig. 8). Accordingly, the hydraulic energy at the crack onset is given by the cumulative volume multiplied by the fluid pressure, i.e., $H_\text{e}=pV_\text{f}$. Finally, a new normalized variable that scales the acoustic emission energy (energy transformed into dynamic wave propagation during failure) with the energy release rate (material resistance to crack propagation) and the hydraulic energy at the fracture onset (input energy during injection) can be written as

23 $$\begin{aligned} {\tilde{\Gamma }}=\frac{\sqrt{G_\text{c}{\bar{E}}}}{H_\text{f}}. \end{aligned}$$

The normalized cumulative acoustic emission energy ${\tilde{\Gamma }}$ is employed to demonstrate the marked transition observed in hydro-fracturing tests at the glass transition temperature.

## Data Availability

The experimental data would be provided upon reasonable request to the corresponding author. The computational data was generated through the open-source solver OpenGeoSys, which is freely available at https://www.opengeosys.org/.
